# 960. Creation of an Infection Prevention and Control (IPAC) Elective for Infectious Disease (ID) Fellows

**DOI:** 10.1093/ofid/ofab466.1155

**Published:** 2021-12-04

**Authors:** John L Kiley, Alice E Barsoumian, Bernadette Thompson, Elizabeth Markelz

**Affiliations:** Brooke Army Medical Center, San Antonio, Texas

## Abstract

**Background:**

ID specialists often function as leaders of IPAC for healthcare systems, with variable training. Our graduates have noted feeling underprepared for this role despite completion of a computer-based training course on IPAC basics. We developed a 2-4 week IPAC elective (IPACe) rotation to address this gap to increase familiarity with key IPAC concepts, introduce learners to approaches to IPAC investigations, and develop understanding of common IPAC challenges and controversies.

**Methods:**

Methodology followed Kern’s 6-step approach. A reading list focusing on key areas in infection prevention was developed. Instructional methods included flipped classroom, learner led discussions, performing tracers, and integration with the IPAC team. Key hospital processes including High Level Disinfection (HLD) and Sterile Processing Department (SPD) were reviewed in detail with and observed by learners. In addition to an IPACe, periodic required IPAC essay questions on real-world investigations as they arose were delivered to the learners. *Learner Assessment*: Learners were assessed on elements of IPAC consistent with the ACGME 6 core competencies at the end of their rotation. *Program Assessment*: Anonymous narrative feedback was solicited post rotation completion and at semi-annual program evaluations. Additionally, learners were asked to rate the elective on a 5 point Likert scale (1 lowest, 5 highest) and specific feedback was solicited for improvement. Finally, feedback was solicited from graduates in IPAC roles.

**Results:**

8 learners participated over from 2017-2021: 2 for 4 weeks, and 6 for 2 weeks. 4 of 8 surveys included a response to the questionnaire, all survey respondents (4/4) rated the elective 5: “rotation should be required of all trainees in the program.” Narrative assessments revealed the elective was highly valuable. Graduates reported feeling well-prepared after the IPACe for their roles as IPAC leaders. Highlights identified were: exposure to interdisciplinary teamwork, participation in tracers in identifying gaps, and using real-world IPAC challenges as cognitive frameworks for outbreak investigation.

**Conclusion:**

An IPACe was highly valued by fellow learners and narrative assessments identified key areas for further focus.

**Disclosures:**

**All Authors**: No reported disclosures

